# Integrated Care in Specialized Networks: Leveraging Early Referrals to Reduce Local Recurrence in Soft Tissue Sarcoma

**DOI:** 10.3390/cancers16213616

**Published:** 2024-10-26

**Authors:** Markus Schärer, Pascale Hösli, Philip Heesen, Georg Schelling, Timothy Obergfell, Kim N. Nydegger, Gabriela Studer, Beata Bode-Lesniewska, Bruno Fuchs

**Affiliations:** 1 Sarcoma Service, Klinik für Orthopädie und Traumatologie, Sarcoma Center, Kantonsspital Winterthur, 8400 Winterthur, Switzerland; 2 Medical Faculty, University of Zurich, 8032 Zurich, Switzerland; 3 Faculty of Health Sciences & Medicine, University of Lucerne, Frohburgstrasse 3, 6002 Luzern, Switzerland; 4 Sarcoma Service, Department of Orthopedics and Trauma, Sarcoma Center, LUKS University Hospital, 6000 Lucerne, Switzerland

**Keywords:** local recurrence (LR), fragmented care pathway (FCP), soft tissue sarcomas (STS), real-world-time data (RWTD), Multidisciplinary Team/Sarcoma Board (MDT/SB), centralized care

## Abstract

This study evaluated the impact of care pathways on the incidence of local recurrence (LR) in patients with soft tissue sarcomas (STS) and identified factors predictive of LR. It compared outcomes between patients managed entirely within a comprehensive care pathway (CCP) at the Swiss Sarcoma Network (SSN) and those who experienced fragmented care pathways (FCPs), where initial treatment occurred outside specialized centers. This prospective study utilized real-world-time data from the SSN-Sarconnector, capturing quality indicators through weekly Multidisciplinary Team/Sarcoma-Board (MDT/SB) meetings. The overall incidence of LR was 17.6% (n = 68/386), higher than rates typically reported in sarcoma center-based studies due to the inclusion of patients with prior inadequate management from real-world referrals. In a univariable logistic regression analysis, the FCP was significantly associated with higher LR rates, unplanned “whoops” resections (25.4%, n = 96), and positive surgical margins, emphasizing the detrimental impact of suboptimal initial management outside of specialized centers. Multivariable analysis confirmed that the FCP (aOR 2.7, 95% CI [1.41, 5.12], *p* = 0.003), tumor size (aOR 1.49, 95% CI [1.1, 2.02], *p* = 0.01), and biological behavior (aOR 5.84 95% CI [1.8, 18.86], *p* = 0.0003) are independent predictors of LR. Notably, patients referred to sarcoma centers after an initial FCP presented with inadequately managed disease, such as incomplete resections and unplanned surgeries, leading to increased complexity of subsequent treatments. These findings underscore the critical role of referral patterns on sarcoma center outcomes, highlighting the significant disparity in LR rates between institutions. The need for improved education and standardized early referral strategies at the spoke level is paramount to optimize patient outcomes and reduce the burden of LR. Enhanced spoke-level education and standardized referral protocols are critical to ensuring effective initial management and optimizing patient outcomes within specialized sarcoma networks like the SSN.

## Simple Summary

This study examines how care pathways impact local recurrence (LR) rates in patients with soft tissue sarcomas (STS). It compares outcomes between those managed entirely within a comprehensive care pathway (CCP) at the Swiss Sarcoma Network (SSN) and those with a fragmented care pathway (FCP) where initial treatment occurred outside specialized centers. Patients in FCPs had higher LR rates, unplanned “whoops” resections, and positive surgical margins, highlighting the critical role of referral patterns and early, coordinated care. The findings underscore the need for better education and standardized early referrals to improve outcomes and establish quality benchmarks in specialized sarcoma care.

## 1. Introduction

The heterogeneous nature of soft tissue sarcomas (STS) presents significant challenges in patient care, often leading to misrecognition and misdiagnosis [1,2]. Due to their rarity and symptom overlap with benign conditions, initial treatments frequently occur outside specialized centers, resulting in inappropriate management, such as unplanned “whoops” resections—excisions performed without prior imaging or biopsy [3,4]. The absence of standardized diagnostic and treatment protocols in non-specialized settings exacerbates outcome variability, underscoring the critical need for management within specialized sarcoma centers [5,6,7]. Treatment guidelines strongly advocate for centralizing care high-volume reference centers where multidisciplinary teams can provide coordinated, guideline-conforming management. This approach significantly reduces the likelihood of unplanned surgeries and has been shown to improve survival rates [2,8,9,10]. This underscores the importance of a coordinated hub-and-spoke model, where specialized sarcoma centers (hubs) guide initial management through timely and accurate referrals from community providers (spokes) [11].

LR, alongside metastasis, serves as a pivotal indicator of disease control and surgical quality [8,12]. LR rates, typically ranging between 7.5 and 15% reflect the presence of residual tumor cells, often due to inadequate resection margins [13,14,15]. Surgery remains the primary treatment modality for STS, aiming for complete control while minimizing functional impairment [16]. The quality of initial surgery was shown to be a major prognostic factor for recurrence-free survival [17]. Therefore, highly trained surgeons in specialized centers are crucial as their expertise in sarcoma surgery and understanding of adjunct treatment options such as radiotherapy can significantly improve resection quality and reduce the need for extensive resections [18,19,20,21]. Preventing LR is critical, as inadequate initial treatments complicate the management of recurrent disease, leading to increased morbidity, higher treatment costs, and poorer patient outcomes [13]. Adhering to treatment guidelines and centralizing care in specialized centers have been shown to reduce LR rates [22,23]. This highlights the necessity of integrated care strategies within specialized centers to balance local control and quality of life effectively. The complex available and constantly renewed treatment strategies need to be based on multidisciplinary care for having the best possible outcome for the patient. This can only be achieved in specialized treatment centers allowing for units of different treatment teams and having the possibility of different treatments.

This study aims to assess the impact of care fragmentation on LR rates in patients treated within the Swiss Sarcoma Network (SSN), specifically evaluating how unplanned “whoops” resections and positive surgical margins contribute to recurrence. Additionally, this study explores how variations in referral patterns across different institutions influence LR outcomes, highlighting the critical role of early and accurate referrals from non-specialized settings. By analyzing the differences between comprehensive and fragmented care pathways, this research seeks to identify potential areas for improvement in patient management to mitigate LR rates. Treatment pathways were analyzed and the differences in comprehensive and fragmented care were assessed. This research aims to serve as a quality assurance benchmark for the SSN, highlighting potential areas for improving patient pathways to mitigate LR rates.

## 2. Materials and Methods

### 2.1. Study Design and SSN

This study uses prospective real-world-time data (RWTD) from patients registered within the SSN-Sarconnector^®^, established in 2018 [24]. The registry acts as a national data warehouse that captures quality indicators related to care pathways, surgical practices, and patient outcomes, all discussed during weekly Multidisciplinary Team/Sarcoma-Board (MDT/SB) meetings. This approach fosters transdisciplinary collaboration and transparent practices in sarcoma therapy, providing a comprehensive data set to assess the impact of care fragmentation on surgical quality. The process of data entry is a collaborative endeavor that engages physicians from diverse disciplines who are integrated into the MDT/SB meetings. It is important to note that in the SSN, patients with sarcoma or suspected sarcoma can enter the process of MDT/SB either through the SSN-Network or directly by the physician, leading in both cases to a final MDT/SB review after central pathology confirmation. The MDT/SB meetings serve as a forum for reviewing patient information, treatment adjustments, and outcomes, thereby ensuring the integrity of the data.

### 2.2. Subjects and Data Extraction

This study included consecutive patients presented to the SSN MDT/SB between 2018 and 2023, either with a suspected diagnosis of ST or as secondary referrals following initial treatment at external institutions. Patients were analyzed based on the date of the first histologically confirmed sarcoma diagnosis and the occurrence of their first LR. Due to the real-world timeline and the inclusion of patients initially managed outside a sarcoma center, the date of diagnosis could precede the study’s inclusion period. Patients treated at a sarcoma center during the 2018–2023 window were followed for the development of LR within this timeframe. Comprehensive data were extracted using the Adjumed platform (Adjumed Services AG, Zurich, Switzerland; accessed on June 2024) including details on patient referrals, treatment modalities, and surgical outcomes.

### 2.3. Definitions, Outcomes, Measurements, and Clinical Characteristics

**Figure 1:**
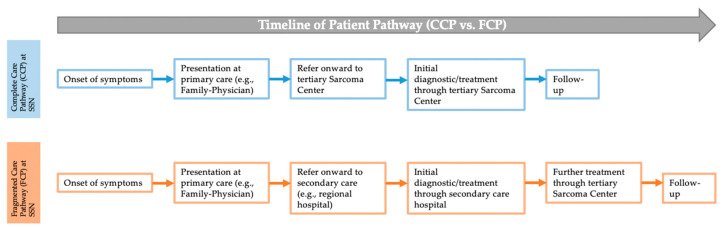
Definition of comprehensive (CTP) and fragmented (FTP) treatment pathway of patient management. The figure illustrates the definition of the different patient pathways: comprehensive care pathway at SSN (CCP) versus fragmented care pathway at SSN (FCP).

Utilizing our RWTD (Adjumed, Zürich, Switzerland), comprehensive demographic and treatment-specific data were systematically extracted and documented for each patient. The variables recorded included age, sex, and treatment institution (1 and 2). In addition, we specifically recorded variables related to the surgical quality and treatment pathway, such as the occurrence of unplanned “whoops” resections, which are closely associated with inadequate surgical margins and increased LR. The patient pathway was categorized into two distinct types: a comprehensive care pathway (CCP) within the SSN; and a fragmented care pathway (FCP), which indicates patients who received initial treatment outside the SSN,—irrespective of the treatment modality—but with subsequent completion in the SSN. The patient pathway is depicted in Figure 1. Furthermore, the tumor’s pathological characteristics were documented, including the biological behavior (intermediate or malignant), and critical dates such as the histological diagnosis, date of the unplanned “whoops” resection, and dates of initial and subsequent treatments (chemotherapy, radiotherapy, or surgery).

Sarcomas of the extremities (affecting both the upper and lower extremities), of the abdomen, including retroperitoneal sarcomas, axial sarcomas (e.g., trunk), head and neck sarcomas as well as sarcomas of the urogenital, perineal, and anal regions were incorporated into this study. Sarcomas were categorized by anatomical compartments (superficial soft-tissue sarcomas (SST-S) and deep soft-tissue sarcomas (DST-S)), and size was assessed in categories (0–50 mm, 51–100 mm, 101–150 mm, and >150 mm) [25]. The surgical excision type was meticulously recorded, differentiating planned resections from unplanned “whoops-resections”. Both neo- and adjuvant therapies were noted. Surgical margins were classified according to Enneking et al. (1980) [26] and Gundle et al. (2018 [27] (R0, R1, R2) and tumor grading (according to Angervall and Kindblom (1993)) [28] (G1, G2, G3) were noted.

### 2.4. Statistical Analysis

Continuous variables are presented as median (interquartile range), while categorical variables are presented as number (percentage). Differences between categorical variables were tested using a Chi-square test or Fisher’s exact test (if the cell frequency was below 5).

A logistic regression analysis was conducted to identify factors associated with LR, focusing on the interplay between care pathways and surgical outcomes. The dependent variable was the presence of LR (yes/no), while independent variables included tumor size, tumor grade, resection margin status, anatomical region, treatment aspects (planned/unplanned (“whoops”) surgery, radiotherapy, or chemotherapy), and the initial treatment pathway (CCP vs. FCP). Univariable logistic regression was first conducted for each independent variable. Variables with a *p*-value < 0.05 in the univariable analysis and possible outcome-affecting variables as well as possible confounding variables were included in the multivariable logistic regression model. The final model for estimating influencing factors for LR included gender, tumor size, tumor grade, biological behavior, anatomic region, compartment, institution, resection margin status, radio-/chemotherapy, and FCP. A *p*-value < 0.05 was considered statistically significant. All analyses were conducted using SAS V.9.4 (SAS Institute, Minato, Japan).

## 3. Results

### 3.1. Study Patient Population

**Figure 2:**
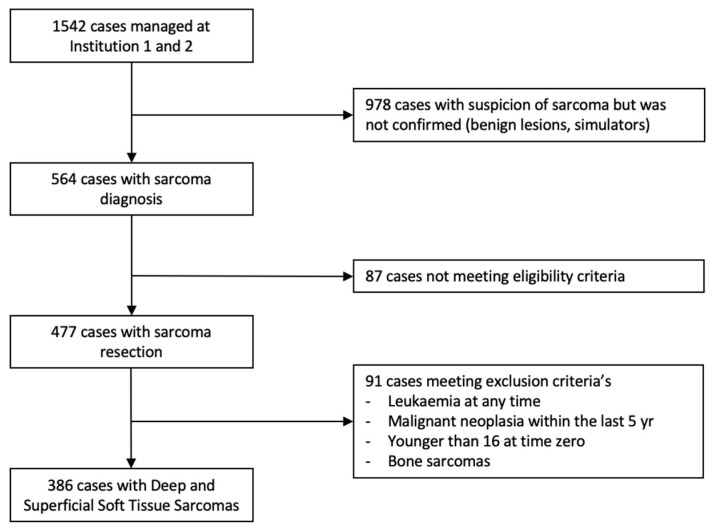
Decision tree on patient inclusion criteria. N, number of patients; SSN, Swiss Sarcoma Network.

Over a 6-year period (2018–2023), a total of 1542 patients with suspected sarcoma were presented to the MDT/SB of the SSN. Of these, 386 patients with an STS were included in the study, focusing on those whose care pathways and surgical outcomes could be clearly linked to comprehensive or fragmented care, as outlined in Figure 2. This distinction is crucial to understanding the impact of care fragmentation on surgical quality and LR rates. Data were analyzed across two tertiary sarcoma centers: Institution 1 managed 40.9% (n = 158) of the patients, while Institution 2 managed 59.1% (n = 228).

**Figure 3:**
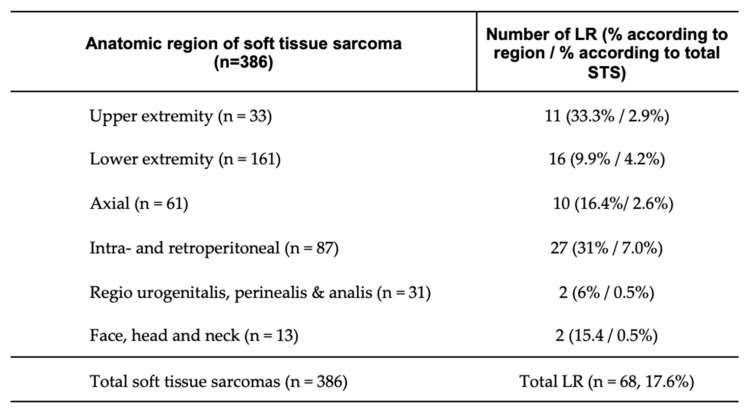
Local recurrence according to anatomical region. Distribution of local recurrence by anatomical region, with data presented as absolute patient numbers and percentages by region, along with percentages relative to the total number of patients with soft tissue sarcoma (STS) in brackets; LR, local recurrence; n, number.

Among the patients analyzed, LR occurred in 17.6% (n = 68). The regional distribution of recurrences, detailed in Figure 3, indicates that the highest recurrence rates were observed in the intra- and retroperitoneal regions at 7.0% (n = 27). Notably, a substantial proportion of these cases involved patients managed via fragmented care pathways (FCPs), underscoring the potential link between FCPs and increased LR.

### 3.2. Characteristics and Factors Associated with Local Recurrence

Factors associated with LR were analyzed differentiating between patient management, tumor characteristics, and treatment-related aspects. The analysis of patient management pathways, FCP versus CCP, revealed that patients with LR were significantly more likely to have been managed through the FCP, with 42.7% (n = 29) compared to 18.9% (n = 60) of patients with non-LR (*p* = 0.0001) (further analysis in the Results Section, Section 3.3).

The comparison of tumor-specific factors between patients with and without LR is summarized in Table 1. LR was significantly more common in patients with DST-S (19.2%, n = 61) compared to SST-S (10.1%, n = 7) (*p* = 0.03, Fisher’s exact test). Furthermore, LR was significantly more frequent in malignant tumors (89.7%, n = 61) compared to those with intermediate biological behavior (70.8%, n = 225; *p* = 0.001), reflecting the increased risk associated with higher-grade tumors often managed through FCPs. Tumors with higher resection grading also showed higher LR rates, with grade 3 resections (G3) being more common in patients with LR (61.2%, n = 41) compared to those without LR (42.2%, n = 133; *p* = 0.006). The abdominal and retroperitoneal regions were most frequently associated with LR (39.7%, n = 27), whereas in non-LR cases tumors were most commonly located in the lower extremity (45.8%, n = 145).

All patients underwent primary surgery, with 74.2% (n = 290) receiving planned surgeries and 25.8% (n = 96) undergoing unplanned “whoops” resections. Notably, whoops resections were associated with positive resection margins in 88.3% (n = 83) of cases, significantly higher than the 22.7% (n = 65) observed in planned resections (*p* < 0.001). This highlights the critical impact of unplanned surgeries, predominantly seen in FCPs, on the quality of surgical margins and subsequent LR risk. There was no significant difference in the occurrence of whoops resections between patients with and without LR; however, positive resection margins were significantly more frequent in patients with LR (53.9%, n = 35) compared to those without LR (35.9%, n = 113; *p* = 0.007) (Table 1).

Radiotherapy was part of the initial treatment in 41.2% (n = 159) of all patients. Among those with LR, 38.2% (n = 26) received radiotherapy during their first treatment cycle, compared to 41.8% (n = 133) in the non-LR group, a difference that was not statistically significant (*p* = 0.58). Chemotherapy was administered in the first treatment cycle to 10.9% (n = 42) of patients, with no significant difference between the LR group (11.8%, n = 8) and the non-LR group (10.7%, n = 34; *p* = 0.8) (Table 1).

In a univariable logistic regression analysis, FCP, tumor size, tumor grade, biological behavior, and positive resection margins were associated with a higher odds of LR (Table A1). In a multivariable logistic regression model, tumor size (adjusted OR 1.49, 95% CI [1.1, 2.02], *p* = 0.01), biological behavior (adjusted OR 5.84 95% CI [1.8, 18.86], *p* = 0.0003), and FCP (adjusted OR 2.7, 95% CI [1.41, 5.12], *p* = 0.003) stayed independently associated with a higher LR (Table A2).

### 3.3. Comparative Analysis of Patient Treatment Pathways: Evaluating Factors Contributing to Increased Risk of LR in FCP vs. CCP

Given the strong association between treatment pathways, particularly the increased risk of LR linked to the FCP, a detailed analysis was conducted to identify contributing factors. Key findings revealed that FCP patients were significantly more likely to undergo unplanned “whoops” resections (50.7%, n = 45) compared to those managed through CCP (17.2%, n = 51; *p* < 0.0001), directly correlating to higher rates of positive resection margins and subsequent LR.

**Figure 4:**
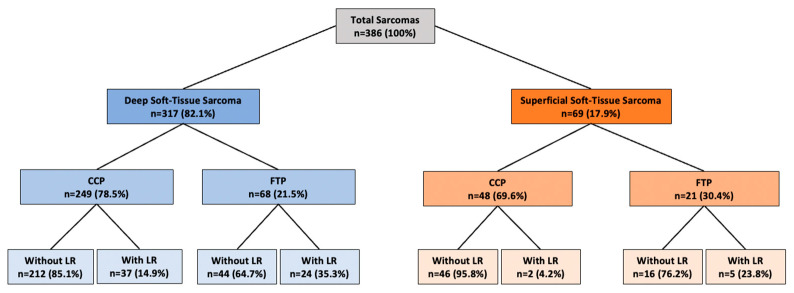
Analysis of treatment pathways (comprehensive vs. fragmented care pathway) for patients presented to the sarcoma board, distinguishing between deep soft-tissue sarcoma and superficial soft-tissue sarcoma, with a focus on local recurrence outcomes. Data presented in numbers (n) and percentage (%); CCP, comprehensive care pathway; FCP, fragmented care pathway; LR, local recurrence; n, number of patients.

Among the 386 patients, 76.9% (n = 297) were treated using the CCP, while 23.1% (n = 89) underwent FCPs (Table 2). The FCP was significantly more common in patients with SST-S compared to DST-S (30.4%, n = 21 vs. 21.5%, n = 68, *p* = 0.04). Figure 4 illustrates the distribution of patients who developed LR, managed through either the FCP or CCP and divided into DST-S and STS-S.

Notably, most SST-S patients treated with the FCP had undergone a whoops resection (85.7%, n = 18) and were subsequently referred to the SSN. Tumor sizes in this group were often between 0 and 50 mm. Comparing further tumor-specific factors between the FCP and CCP, a higher proportion of patients with the FCP had malignant disease (82%, n = 73) compared to those with the CCP (71.7%, n = 213). Initial tumor size and tumor grading as well as treatment-specific aspects such as radio-/chemotherapy in the primary treatment plan did not differ significantly between the two patient groups (Table 2).

Notably, patients with the FCP had significantly more frequent whoops resections compared to those with the CCP (50.7%, *n* = 45 vs. 17.2%, *n* = 51; *p* < 0.0001). In addition, significantly more often positive resection margins were observed in patients treated by FCPs compared to CCPs (61.8%, n = 55 vs. 31.3%, n = 93; *p* = 0.0001). Patients with the FCP were managed significantly more often at Institution 1 compared to those with the CCP (59.6%, *n* = 53 vs. 35.4%, *n* = 105; *p* = 0.001) (Table 2).

**Figure 5:**
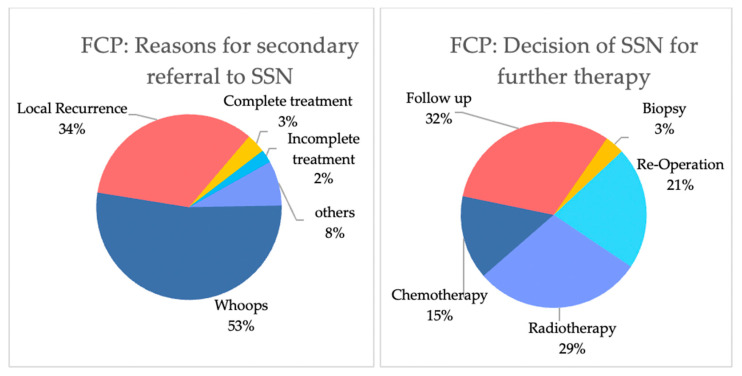
Patients with a fragmented care pathway: reasons for secondary referral towards a sarcoma hub (**left**) and decision of the sarcoma board (**right**). On the left, other reasons include metastasis or requests for a second opinion. “Complete treatment outside SSN” refers to patients treated outside a sarcoma hub but referred for follow-up at a sarcoma hub. “Incomplete treatment outside” includes patients whose treatment was not completed outside the sarcoma hub. (**Right**) Decisions and treatments determined by the sarcoma board.

Figure 5 outlines the reasons why patients were initially treated by FCPs and the decisions at the subsequent sarcoma board. The primary reasons for referral were whoops resection (52.8%, n = 47) and LR (33.7%, n = 30). Following referral, the SSN predominantly recommended further interventions, including re-operation (21.4%, n = 19), radiotherapy (29.2%, n = 26), or chemotherapy (14.6%, n = 13).

The impact of the FCP was evaluated using univariable logistic regression analysis, which indicated associations between the FCP and sarcoma center institution, positive resection margins, LR, and whoops resections (Table A3). Multivariable logistic regression analysis revealed independent associations between the FCP and whoops resections (adjusted OR = 6.62, 95% CI [2.99, 14.7], *p* < 0.0001), specific sarcoma center institution (adjusted OR = 0.32, 95% CI [0.18, 0.56], *p* < 0.0001), and LR (adjusted OR = 2.96, 95% CI [1.5, 5.79], *p* = 0.001). Details are presented in Table A4.

### 3.4. Differences Between Dedicated Sarcoma Centers

Significant differences were observed between the dedicated sarcoma referral networks, with Institution 1 managing a higher percentage of patients with LR (20.9%) compared to Institution 2 (15.3%, *p* < 0.0001). This disparity reflects variations in referral patterns and the prevalence of FCP, where Institution 1 had a notably higher proportion of patients managed through FCP (33.5% vs. 15.8%, *p* < 0.0001). Our data suggest that the initial quality of care at the spoke level, represented by these referral patterns, plays a decisive role in patient outcomes. Despite subsequent management at the specialized sarcoma hubs, initial fragmented care contributed significantly to unplanned surgeries, inadequate resection margins, and increased LR rates, underscoring the importance of optimizing referral quality and pathways at the spoke level.

Tumor-specific factors were similar across the two institutions, with no statistical significance. Additionally, there were no differences regarding the use of radiotherapy (Institution 1: 39.2%, Institution 2: 52.5%, *p* = 0.55) and chemotherapy (Institution 1: 12.0%, Institution 2: 10.1%, *p* = 0.55) as part of the first treatment cycle. The rates of planned versus whoops resections were also similar (Institution 1: 26.0%, Institution 2: 24.1%, *p* = 0.68). tumor grading, initial size, and positive resection margins were comparable and statistically not significant between the institutions. Further details are presented in Table 3.

## 4. Discussion

This study highlights the critical importance of centralized care in reducing LR rates, minimizing the incidence of unplanned “whoops” resections, and ensuring access to comprehensive adjunctive treatments. Importantly, the findings underscore a key aspect of healthcare delivery: the significant impact of referral patterns on sarcoma center outcomes. The disparity in LR rates between institutions reflects not only the challenges faced by centers in compensating for prior inadequate care but also underscores the need for better education and early referral strategies at the spoke level. This represents a pivotal area for health service improvement as optimizing referral practices could significantly reduce LR rates and improve overall patient outcomes.

Our findings reveal that FCPs are significantly associated with increased LR rates, primarily driven by the higher incidence of unplanned “whoops” resections and the resulting positive resection margins. These findings underscore the detrimental effects of initial management outside of specialized centers and highlight the crucial role of coordinated, guideline-conforming care in specialized settings.

Our findings underscore the critical role of referral quality in the hub-and-spoke model of sarcoma care. The significant disparity in LR rates between the two institutions directly correlates with the prevalence of FCP, indicating that initial management at the spoke level is a primary determinant of long-term outcomes [8,10,29]. Even when patients are subsequently managed within specialized hubs, the damage from initial inadequate care often remains irreversible, as evidenced by the higher rates of unplanned surgeries and positive resection margins in FCP patients. This highlights a pivotal area for health service improvement: enhancing the education and protocols at the spoke level to ensure timely and accurate referrals to specialized hubs, thereby reducing LR risks and improving surgical outcomes. These findings advocate for a strategic approach that integrates care pathways across the entire sarcoma network, reinforcing the importance of comprehensive care from the outset.

Our study reported an overall LR rate of 17.6%, which is slightly higher than the 7–15% LR rates typically reported in studies conducted within sarcoma centers [13,14,15]. This discrepancy underscores the adverse impact of care fragmentation, as a substantial proportion of our cohort included patients initially treated outside these centers, reflecting the critical influence of the initial care setting on long-term outcomes (and inclusion of all real-world referrals of a specialized sarcoma network). We identified a strong correlation between LR and factors such as care pathways (CTP vs. FCP), tumor size, biological behavior, and resection margins, consistent with previous research [14,30]. Notably, the FCP emerged as a significant independent predictor of LR, further validating the critical impact of treatment fragmentation on patient outcomes. According to both the European Society for Medical Oncology (ESMO) and the National Comprehensive Cancer Network (NCCN), multidisciplinary management is mandatory for all sarcoma patients [2,16]. This approach has been validated by large-scale studies, such as those by Voss et al. and Bonvalot et al., which demonstrated survival advantages for patients treated in specialized centers [10,29]. LR poses substantial clinical challenges, frequently necessitating complex reoperations and, in severe cases, resulting in significant tissue loss or limb amputation [13,15]. These severe consequences underscore the need for stringent adherence to centralized, high-standard care from the outset to minimize the risk of LR and its associated complications. Despite the well-established benefits, our study found that 23.1% (n = 89) of patients were still treated via FCPs, which multivariable logistic regression analysis confirmed as an independent risk factor for LR.

Our evaluation of FCP patients revealed several distinguishing characteristics, including a higher prevalence of unplanned “whoops” resections, positive resection margins, LR, and aggressive biological tumor behavior. These findings highlight the compounded risks associated with FCPs and underscore the necessity for early referral to specialized centers to mitigate these risks. By contrast, patients managed through the CTP experienced significantly fewer whoops resections and fewer positive resection margins. Both resection margins and whoops resections play a critical role in LR, as data of the SSN showed that whoops resections are associated with reduced local-recurrence-free survival [31]. These two parameters, resection margins and whoops resections, are also important benchmarks of surgical quality in sarcoma treatment [2,18,32,33].

Secondary referrals to sarcoma centers most frequently stemmed from whoops resections (53%) or LR (34%). Many of these patients required reoperations (21%) or additional radiotherapy (29%) or chemotherapy (15%). Surgical quality in sarcoma care is closely tied to the surgeon’s expertise and specialization [2]. While wide excisions with negative margins are the standard treatment, complex tumor anatomy may necessitate individualized excision strategies and additional treatments, underscoring the need for a multidisciplinary approach [2,9]. Centralized sarcoma care delivers superior surgical and overall outcomes, driven largely by the collaborative expertise of multidisciplinary teams, including radiologists, pathologists, oncologists, and specialized surgeons, working in concert to optimize patient care [2,9]. Our study highlights the essential role of these teams, particularly in the management of rare and complex diseases like sarcoma.

Importantly, our study identified a significant correlation between FCPs and the specific sarcoma centers, suggesting regional disparities in referral patterns and access to specialized care. This variation may contribute to inconsistent patient outcomes, highlighting the need for standardized referral practices and improved access to centralized care across all regions. One center managed a higher proportion of FCP patients and had more frequent cases of recurrent disease. Notably, tumor-specific factors such as size, resection margins, and grading did not significantly differ across centers, indicating that regional differences in healthcare infrastructure and referral practices may contribute to varying LR rates. Centers that manage patients from the point of diagnosis benefit from a more consistent and comprehensive treatment approach, while those receiving patients after initial treatment elsewhere may face difficulties due to inadequate early management. These findings underscore the importance of early and consistent referral to specialized sarcoma centers. Once the damage from initial treatment outside these centers occurs, it may not be fully correctable. Standardizing referral practices and improving access to centralized sarcoma care are essential to mitigating regional disparities and optimizing treatment outcomes across different regions.

While our study primarily focused on the impact of care pathways on local recurrence (LR) rates in soft tissue sarcoma (STS) patients, it is crucial to consider whether these differences translate into variations in overall survival (OS) and disease-specific survival (DSS). The relationship between care pathways and survival outcomes is complex and influenced by multiple confounding factors, including tumor size, grade, anatomical location, and biological behavior. Previous research, including our Target Trial Emulation (TTE) study [31], has shown that unplanned resections—more prevalent in fragmented care pathways (FCPs)—increase the risk of LR but do not significantly affect metastasis-free survival (MFS) or OS when compared to planned resections. This may be attributed to the fact that unplanned resections often involve smaller, superficial tumors with less aggressive histology, particularly in the upper extremities. These tumors, despite higher LR rates due to inadequate initial management, inherently have a better prognosis concerning distant metastasis and survival outcomes. Conversely, patients undergoing planned resections within comprehensive care pathways (CCPs) are more likely to present with larger, higher-grade tumors at a more advanced stage [34]. These factors inherently increase the risk of distant metastasis and negatively impact OS and DSS. Therefore, while CCPs are associated with better local control, the overall prognosis is predominantly determined by tumor biology and stage at presentation rather than the care pathway alone. Analyzing survival outcomes between CCP and FCP was beyond the scope of this study due to the complexity of adjusting for these confounding variables in a real-world data setting. Meaningful comparisons would require sophisticated methodologies, such as predictive modeling and TTE approaches, to account for the myriad of factors influencing survival. Our ongoing research is dedicated to exploring these aspects in greater detail, aiming to provide a more comprehensive understanding of how care pathways influence long-term patient outcomes. These insights underscore the paramount importance of early and accurate diagnosis—“predictive diagnostics”—and timely referral to specialized centers. By facilitating appropriate management strategies at an earlier stage, especially for aggressive tumors, we can not only improve local control but potentially enhance overall survival outcomes. This aligns with our findings emphasizing the critical role of integrated care strategies within specialized networks like the Swiss Sarcoma Network (SSN).

This study has several limitations that warrant consideration in interpreting findings. One of the primary limitations is the potential for selection bias due to the referral process. Patients referred secondarily to the SSN are more likely to represent complex cases, often involving complications or suboptimal management, while those with good outcomes outside the SSN may not be presented at all. This may lead to an overrepresentation of complex cases, potentially skewing the findings towards higher rates of LR and complications. These limitations underline the need for studies to further validate the impact of care pathways on LR in soft tissue sarcoma patients.

The generalizability of the findings may also be limited as the cohort is drawn exclusively from Switzerland, where healthcare systems and referral patterns may differ from those in other countries. Moreover, focusing on two main institutions may not fully represent the care provided across all centers within the SSN, introducing potential institutional bias. Lastly, incomplete patient records and varying follow-up durations could influence the observed LR rates, potentially weakening the conclusions regarding the impact of comprehensive versus fragmented care pathways.

## 5. Conclusions

In conclusion, our study demonstrates that the FCP is significantly associated with higher LR rates in STS patients. While FCPs increase the risk of LR primarily due to unplanned “whoops” resections and positive resection margins, the OS and DSS outcomes appear to be more closely linked to tumor biology and stage of presentation rather than the care pathway alone. The variability in referral practices across institutions significantly impacts patient outcomes, underscoring the need for a cohesive and integrated approach within the hub-and-spoke model of sarcoma care. Enhancing education, standardizing referral protocols, and ensuring early referral at the spoke level are strategic imperatives to mitigate LR risks and optimize patient outcomes across specialized networks like the Swiss Sarcoma Network (SSN). These findings highlight the necessity of a comprehensive, coordinated care strategy that transcends individual institutions and addresses the full continuum of sarcoma management, from early diagnosis to long-term follow-up.

## Figures and Tables

**Table 1:** Assessment of patient-specific tumor characteristics and treatment aspects between patients with and without local recurrence.

Characteristics	Overall (%)	No Local Recurrence (%)	Local Recurrence (%)	*p*-Value
n, (%)	386	318 (82.4)	68 (17.6)	
Female, n (%)	186 (48.2)	154 (82.8/48.4)	32 (17.2/47.1)	0.84
Compartment				
DST-S	317 (82.1)	256 (80.8/80.5)	61 (19.2/89.7)	0.03
SST-S	69 (17.9)	62 (89.9/19.5)	7 (10.1/10.3)	
Institution				0.16/0.04 ^a^
1	158 (40.9)	125 (79.1/39.3)	33 (20.9/48.5)	
2	228 (59.1)	193 (84.6/60.6)	35 (15.4/51.5)	
Patient pathway				0.0001
CCP	297 (76.9)	258 (86.9/81.1)	39 (13.1/57.4)	
FCP	89 (23.1)	60 (67.4/18.9)	29 (32.6/42.7)	
Biological behavior				0.001
Malignant	286 (74.1)	225 (78.7/70.8)	61 (21.3/89.7)	
Intermediate	100 (25.9)	93 (93.0/29.3)	7 (7.0/10.3)	
Region, (%)				0.0001
Face, Head, Neck	13 (3.4)	11 (84.6/3.5)	2 (15.4/2.9)	0.29
Upper extremity	33 (8.6)	22 (66.6/6.9)	11 (33.3/16.2)	0.01
Lower extremity	161 (41.7)	145 (90.1/45.8)	16 (9.9/23.5)	0.0008
Axial	61 (15.8)	51 (83.6/16.0)	10 (16.4/14.7)	0.79
Intra- and retroperitoneal	87 (22.5)	60 (69.0/18.9)	27 (31.0/39.7)	0.0002
Urogenital, perineal, and anal regions	31 (8.0)	29 (93.5/9.1)	2 (6.5/2.9)	0.09
Initial Size, mm				0.12
0–50 mm	108 (28.0)	94 (84.3/29.6)	14 (15.7/20.6)	0.14
51–100 mm	133 (34.5)	111 (83.5/34.9)	22 (16.5/32.4)	0.69
101–150 mm	74 (19.2)	61 (82.4/19.2)	13 (17.6/19.1)	0.99
>150 mm	71 (18.4)	52 (73.2/16.4)	19 (26.8/27.9)	0.03
Tumor grading	^d^	^c^	^b^	0.01
G1	138 (36.1)	123 (89.1/39.1)	15 (10.9/22.4)	0.009
G2	70 (18.3)	59 (84.3/18.7)	11 (15.7/16.4)	0.64
G3	174 (45.6)	133 (76.4/42.2)	41 (23.6/61.2)	0.006
Resection margin	^e^	^c^	^c^	0.02
R0 wide margin/R0	232 (61.1)	202 (87.1/64.1)	30 (12.9/46.2)	0.003
R1 marginal margin/R1	122 (32.1)	92 (75.4/29.2)	30 (24.6/46.2)	0.01
R2 intralisional margin/R2	26 (6.8)	21 (80.8/6.7)	5 (19.2/7.7)	0.2
Radiotherapy part of first treatment	159 (41.2)	133 (83.7/41.8)	26 (16.3/38.2)	0.58
Chemotherapy as part of first treatment	42 (10.9)	34 (81.0/10.7)	8 (19.0/11.8)	0.8
Whoops resection	96 (25.8)	79 (82.3/24.8)	17 (17.37/25.0)	0.94

Data presented in numbers (n) of patients, with percent values in brackets (according to the total overall of the specific variable/according to non-local recurrence or local recurrence patients). Patients are categorized by the presence or absence of local recurrence. *p*-values indicate statistical significance of differences between groups, with a threshold of <0.05 considered significant. If two *p*-values are provided, the first *p*-value is calculated by Chi-square test and the second indicated by Fisher’s exact test. Biological behavior was defined according to the WHO criteria into malignant and intermediate, and tumor grading according to Angervall and Kindblom (1993) [28]. Resection margins were defined according to Enneking et al. (1980) [26] (R0, R1, R2). DST-S, deep soft-tissue sarcoma; IQR, interquartile range; n, number; SST-S, superficial soft-tissue sarcoma. ^a^ *p*-value calculated by Fisher’s exact test ^b^ one patient not classified; ^c^ three patients not classified; ^d^ four patients not classified, ^e^ six patients missing.

**Table 2:** Comparison of patient characteristics based on the different treatment pathways: comprehensive care pathway providing full treatment by a sarcoma center (CCP) vs. fragmented care pathway with initial care outside a dedicated sarcoma center (FCP).

Characteristics	Overall (%)	CCP (%)	FCP (%)	*p*-Value
n, (%)	386	297 (76.9)	89 (23.1)	na
Female, n (%)	186 (48.2)	139 (74.7/46.8)	47 (25.3/52.8)	0.32
Institution				
1	158 (40.9)	105 (66.5/35.4)	53 (33.5/59.6)	0.001
2	228 (59.1)	192 (84.2/64.7)	36 (15.8/40.5)	
Biological behavior				0.05
Malignant	286 (74.1)	213 (74.5/71.7)	73 (25.5/82.0)	
Intermediate	100 (25.9)	84 (84.0/28.3)	16 (16.0/18.0)	
Compartment				0.04
DST-S	317 (82.1)	249 (78.5/83.8)	68 (21.5/76.4)	
SST-S	69 (17.9)	48 (69.6/16.2)	21 (30.4/23.6)	
Region, n %				0.02
Face, Head, Neck	13 (3.4)	10 (76.9/3.4)	3 (23.1/3.4)	0.99
Upper extremity	33 (8.6)	19 (57.6/6.4)	14 (42.4/15.7)	0.006
Lower extremity	161 (41.7)	134 (83.2/45.1)	27 (16.8/30.3)	0.01
Axial	61 (15.8)	45 (73.8/15.2)	16 (26.2/18.0)	0.52
Intra- and retroperitoneal	87 (22.5)	62 (71.3/20.9)	25 (28.7/28.1)	0.15
Urogenital, perineal, and anal regions	31 (8.0)	27 (87.1/9.1)	4 (12.9/4.5)	0.16
Initial Size, mm				0.6
0–50 mm	108 (28.0)	81 (75.0/27.3)	27 (25.0/30.3)	0.57
51–100 mm	133 (34.5)	99 (74.4/33.3)	34 (25.6/38.2)	0.4
101–150 mm	74 (19.2)	59 (79.7/19.9)	15 (20.3/16.9)	0.53
>150 mm	71 (18.4)	58 (81.7/19.5)	13 (18.3/14.6)	0.29
Tumor grading	^c^	^b^	^a^	0.78
G1	138 (36.1)	106 (76.8/36.1)	32 (23.2/36.4)	0.96
G2	70 (18.3)	56 (80.0/19.1)	14 (20.0/15.9)	0.5
G3	174 (45.6)	132 (75.9/44.9)	42 (24.1/47.7)	0.65
Resection margin	^e^	^d^	^a^	0.001
R0 wide margin/R0	232 (61.1)	199 (85.8/68.2)	33 (14.2/37.5)	0.0001
Positive resection margins (R1 + R2)	148 (38.9)	93 (62.8/31.3)	55 (37.2/61.8)	0.0001
Chemotherapy part of first treatment	42 (10.9)	33 (78.6/11.1)	9 (21.4/10.1)	0.08
Radiotherapy part of first treatment	159 (41.2)	128 (80.5/43.1)	31 (19.5/34.8)	0.9
Whoops resections	96 (24.8)	51 (53.1/17.2)	45 (46.9/50.6)	<0.0001
Local recurrence n, %	68 (17.6)	39 (57.4/13.1)	29 (43.6/32.6)	<0.0001

Data presented in numbers (n) of patients, with percent values in brackets (according to the total overall of the specific variable/according to comprehensive or fragmented care pathway). Patients are categorized by comprehensive or fragmented care pathway. *p*-values indicate statistical significance of differences between groups, with a threshold of <0.05 considered significant. Biological behavior was defined according to the WHO criteria into malignant and intermediate and tumor grading according to Angervall and Kindblom (1993) [28]. Resection margins were defined according to Enneking et al. (1980) [26] (R0, R1, R2). DST-S, deep soft-tissue sarcoma; IQR, interquartile range; n, number; SST-S, superficial soft-tissue sarcoma. ^a^ one patient not classified; ^b^ three patients not classified; ^c^ four patients not classified, ^d^ five patients missing; ^e^ six patients missing.

**Table 3:** Analysis of patient, tumor, treatment aspects, and local recurrence addressing regional referral differences through a comparison of the sarcoma centers.

Characteristics	Overall (%)	Sarcoma Center Institution 1 (%)	Sarcoma Center Institution 2 (%)	*p*-Value
n, (%)	386	158 (40.9)	228 (59.1)	
Female, n (%)	186 (48.2)	71 (38. 2/44.9)	115 (61.8/50.4)	0.29
Treatment pathway				0.0001
CCP	297 (76.9)	105 (35.4/66.5)	192 (64.6/84.2)	
FCP	89 (23.1)	53 (59.6/33.5)	36 (40.4/15.8)	
Biological behavior				0.63
Malignant	286 (74.1)	115 (40.2/72.8)	171 (59.8/75.0)	
Intermediate	100 (25.9)	43 (43.0/27.2)	57 (57.0/25.0)	
Compartment				0.74
DST-S	317 (82.1)	131 (41.3/82.9)	186 (58.6/81.6)	
SST-S	69 (17.9)	27 (39.1/17.1)	42 (60.9/18.4)	
Initial Size, mm (IQR)	80 (50–130)	81 (50–130)	80 (50–133)	0.8
Tumor grading	^d^	^a^	^a^	
G1	138 (36.1)	62 (44.9/39.2)	76 (55.1/33.3)	0.23
G2	70 (18.3)	30 (42.9/19.0)	40 (57.1/17.5)	0.72
G3	174 (45.6)	64 (36.8/40.5)	110 (63.2/48.3)	0.13
Whoops resections	96 (24.8)	41 (42.7/26.0)	55 (57.3/24.1)	0.68
Positive resection margins	148 (39.0) ^c^	68 (45.9/43.7) ^b^	80 (54.1/35.6) ^b^	0.1
Local recurrence n, %	68 (17.6)	33 (48.5/20.9)	35 (51.5/15.3)	0.0001

Data presented in numbers (n) of patients, with percent values in brackets (according to the total overall of the specific variable/according to the sarcoma center institution 1 or 2). Patients are categorized into groups of treatment by sarcoma center 1 or 2. *p*-values indicate statistical significance of differences between groups, with a threshold of <0.05 considered significant. ^a^ two patients not classified; ^b^ three patients not classified; ^c^ six patients missing; ^d^ four patients not classified.

## Data Availability

The data presented in this study are available on request from the corresponding author.

## Appendix A

**Table A1:** Univariable logistic regression analysis for predictors of local recurrence in soft tissue sarcoma.

Variable	Local Recurrence		
	OR	95% CI	*p*-Value
Gender	0.95	(0.56, 1.6)	0.84
Institution	0.69	(0.41, 1.16)	0.16
FCP	3.2	(1.83, 5.58)	<0.0001
Compartment	0.48	(0.21, 1.09)	0.08
Tumor size	1.32	(1.04, 1.69)	0.03
Tumor grading	1.6	(1.16, 2.19)	0.004
Biological behavior	3.6	(1.59, 8.17)	0.002
Anatomic region	1.09	(0.88, 1.35)	0.41
Positive resection margins	2.09	(1.22–3.58)	0.008
Radiotherapy part of first treatment	0.86	(0.5, 1.47)	0.59
Chemotherapy part of first treatment	1.11	(0.49, 2.53)	0.8
Whoops resection	1.01	(0.6, 1.85)	0.9

Univariable logistic regression analysis: data are odds ratios (ORs) with 95% confidence interval (CI) in brackets from univariable logistic regression analysis; OR, odds ratio; 95% CI, 95% Confidence interval. *p*-value < 0.05 was defined as statistically significant.

**Table A2:** Multivariable logistic regression analysis results for predictors of local recurrence in soft tissue sarcoma.

Variable	Local Recurrence		
	aOR	95% CI	*p*-Value
Gender	1.004	(0.56, 1.8)	0.99
Institution	0.9	(0.49, 1.6)	0.72
Compartment	0.47	(0.18, 1.24)	0.13
FCP	2.7	(1.41, 5.12)	0.003
Biological behavior	5.84	(1.8, 18.86)	0.003
Size	1.49	(1.1, 2.02)	0.01
Anatomic region	0.89	(0.69, 1.16)	0.4
Tumor grading	1.28	(0.85, 1.9)	0.23
Positive resection margins	1.8	(0.99, 3.35)	0.05
Radiotherapy part of first treatment	0.55	(0.29, 1.05)	0.07
Chemotherapy part of first treatment	0.77	(0.29, 1.88)	0.57

Multivariable logistic regression analysis: data showing adjusted odds ratio with 95% Confidence interval in brackets and *p*-values for the different variables in the model. aOR, adjusted odds ratio; FCP, fragmented care pathway; 95% CI, 95% confidence interval. *p*-value < 0.05 was defined as statistically significant.

**Table A3:** Univariable logistic regression analysis for fragmented care pathway.

Variable	Fragmented Care Pathway		
	OR	95% CI	*p*-Value
Gender	1.27	(0.79, 2.04)	0.32
Institution	0.37	(0.23, 0.6)	<0.0001
Compartment	1.6	(0.9, 2.86)	0.11
Tumor size	0.87	(0.69, 1.09)	0.22
Tumor grading	1.03	(0.79, 1.35)	0.82
Biological behavior	1.8	(0.99, 3.27)	0.05
Anatomic region	0.96	(0.79, 1.17)	0.69
Resection margins	3.57	(2.17, 5.86)	<0.0001
Radiotherapy as part of first treatment	0.96	(0.6, 1.54)	0.86
Chemotherapy as part of first treatment	0.75	(0.95, 2.68)	0.08
Local recurrence	3.2	(1.83, 5.56)	<0.0001
Whoops resection	4.93	(2.95, 8.24)	<0.0001

Univariable logistic regression analysis: data are odds ratios (ORs) with 95% confidence interval (CI) in brackets from univariable logistic regression analysis; OR, odds ratio; 95% CI, 95% confidence interval. *p*-value < 0.05 was defined as statistically significant.

**Table A4:** Multivariable logistic regression analysis for the associations between tumor, patients, treatment characteristics, and the fragmented care pathway.

Variable	Fragmented Care Pathway		
	aOR	95% CI	*p*-Value
Gender	1.57	(0.9, 2.74)	0.1
Institution	0.32	(0.18, 0.56)	<0.0001
Tumor size	1.21	(0.89, 1.63)	0.22
Biological behavior	2.61	(1.1, 6.4)	0.04
Tumor grading	0.9	(0.61, 1.32)	0.58
Positive resection margins	1.4	(0.71, 2.79)	0.34
Local recurrence	2.96	(1.5, 5.79)	0.001
Whoops resection	6.62	(2.99, 14.7)	<0.0001

Multivariable logistic regression analysis: data showing adjusted odds ratio with 95% confidence interval in brackets and *p*-values for the different variables in the model. aOR, adjusted odds ratio; 95% CI, 95% confidence interval. *p*-value < 0.05 was defined as statistically significant.
